# A Community-Based Event Delivery Protocol in Publish/Subscribe Systems for Delay Tolerant Sensor Networks

**DOI:** 10.3390/s91007580

**Published:** 2009-09-28

**Authors:** Nianbo Liu, Ming Liu, Jinqi Zhu, Haigang Gong

**Affiliations:** Department of Computer Science and Engineering, University of Electronic Science and Technology of China, Cheng du 610054, China; E-Mails: liunb@uestc.edu.cn (N.L.); jingpei719@163.com (J.Z.)

**Keywords:** event delivery, publish/subscribe, queue management, community

## Abstract

The basic operation of a Delay Tolerant Sensor Network (DTSN) is to finish pervasive data gathering in networks with intermittent connectivity, while the publish/subscribe (Pub/Sub for short) paradigm is used to deliver events from a source to interested clients in an asynchronous way. Recently, extension of Pub/Sub systems in DTSNs has become a promising research topic. However, due to the unique frequent partitioning characteristic of DTSNs, extension of a Pub/Sub system in a DTSN is a considerably difficult and challenging problem, and there are no good solutions to this problem in published works. To ad apt Pub/Sub systems to DTSNs, we propose CED, a community-based event delivery protocol. In our design, event delivery is based on several unchanged communities, which are formed by sensor nodes in the network according to their connectivity. CED consists of two components: event delivery and queue management. In event delivery, events in a community are delivered to mobile subscribers once a subscriber comes into the community, for improving the data delivery ratio. The queue management employs both the event successful delivery time and the event survival time to decide whether an event should be delivered or dropped for minimizing the transmission overhead. The effectiveness of CED is demonstrated through comprehensive simulation studies.

## 1. Introduction

The traditional sensor network is composed of a large number of densely deployed sensor nodes with short range radio and several sink nodes, and sensors in the network collaborate together to collect the target data and transmit them to the sink nodes. This approach, however, may not work effectively in scenarios with extremely low and/or intermittent connectivity due to sparse network density, obstacles, sensor node mobility or sensor energy exhaustion and so on. For example, in wild animal studies, researchers often install static sensors at some watering places and drive vehicles as mobile nodes to visit these disjoint sensors for data collection as a cost-efficient solution. Accordingly, the delay tolerant sensor network (DTSN) has been recently proposed. DTSNs belong to the general category of Delay Tolerant Networks (DTNs) [1], that is, networks will incur delays that can be very large and unpredictable. A DTSN is characterized by sensor nodes' intermittent connectivity. That is, it is difficult to form a well connected end-to-end path for all the sensor nodes to transmit data in the network.

The publish/subscribe system is used to connect the distributed information providers and consumers in an asynchronous way, where there are subscribers, publishers and brokers [2]. The subscribers show their interest in certain events by submitting predefined subscriptions; the publishers issue newly detected events to the system; the brokers, which are generally custom servers, collect those subscriptions and events, match them and notify the subscribers of the matched events. In this way, the system supports loosely coupled interactions, such as activity monitoring systems.

Much research has been done in the context of designing Pub/Sub systems in wireless networks [3,4], where the authors all assume that the backbone network is still wired and connective. However, since a DTSN is quite different from the traditional wireless sensor network due to the particular sensor node characteristics, i.e., intermittent connectivity and limited energy, those previous Pub/Sub communication solutions are not suitable for DTSNs, and how to extend Pub/Sub systems in DTSNs is a challenge.

In this paper, we propose a community-based event delivery protocol (CED) that adapts Pub/Sub systems to DTSNs. In our design, event delivery is based on several unchanged communities, which are formed by sensors in the network according to their connectivity. CED consists of two components for event delivery and queue management. In event delivery, events in a community are delivered to mobile subscribers once a subscriber comes into the community, for improving the data delivery ratio. The queue management employs both the event successful delivery time and the event survival time to decide whether the event should be transmitted or dropped for minimizing the transmission overhead. We evaluate the performance of the proposed approach by doing simulation and comparing it with the direct gathering protocol (DG). Simulation results show that our approach achieves a higher data delivery ratio with the lower transmission overhead and delivery delay than DG. To our best knowledge, our design is the first solution for DTSN, in the sense that it is the first attempt to apply communities in Pub/Sub systems for DTSN and use subscriber mobility prediction to support continuous event delivery in Pub/Sub systems.

Our design addresses the following questions: (1) how to divide the network into several communities? (2) How to implement event delivery based on the constructed communities? (3) Why and how to design the event queue management scheme?

The rest of the paper is organized as follows. Section 2 provides an overview of related work, while Section 3 presents the mobility model and basic assumptions. In Section 4, we introduce CED step by step. Section 5 shows the effectiveness of CED via simulation. Finally, Section 6 concludes the paper.

## 2. Related Works

In this section, we classify research on routing strategies in Pub/Sub systems into three categories: routing strategies for traditional mobile networks, routing strategies in Pub/Sub systems for mobile *ad hoc* networks (MANET) and routing strategies in Pub/Sub systems for DTN, respectively.

### 2.1. Routing Strategies in Pub/Sub Systems for Traditional Mobile Networks

In mobile networks, the link between any two nodes is not as stable as in fixed networks. To adapt Pub/Sub systems to mobile environments, several strategies are proposed. Sutton *et al.* [5] proposed the use of a central proxy to tackle subscriptions for disconnected mobile clients. After reconnecting to the system, a mobile client will first connect to the central proxy to obtain the events published during their absence. The central proxy, however, tends to become a performance bottleneck and the system does not have good scalability.

In [6], Podnar and Lovrek discuss a persistent notification protocol to support mobile clients in a Pub/Sub system. In this approach, every broker keeps a list of the IDs for the events published by the broker itself and buffers the published events according to their lifetime. When a mobile client connects a new broker, the client will submit the IDs of the latest events received by it to the new broker. The new broker will search the new events for the client in the whole system.

Burcea *et al.* [7] deploy a simple handover protocol based on the destination prediction of mobile clients. However, their work has ignored the problem of continuous event delivery for a client during its movement and this problem is more challenging than the simple client movein/moveout problem solved in their work. The mobility of publishers has been discussed in [8]. The authors proposed four solutions to alleviate the impact of publisher's mobility on the performance of the Pub/Sub systems. Moreover, there are also studies in [9,10] concerning the reliability in event transmission of mobile clients in Pub/Sub systems.

### 2.2. Routing Strategies in Pub/Sub Systems for MANET

In recent years, many works [11,12] have been developed Pub/Sub systems in mobile *ad hoc* networks. For example, in [11], Baldoni *et al.* propose a structure-less routing protocol, where a distributed implementation of the dispatching service is realized by running a broker on each mobile node of the MANET. Unlike traditional cases, that paper leverages off the broadcast communications available in a MANET to forward events to multiple destinations and lets each receiving broker autonomously decide if and when re-forwarding the event on the basis of an estimation of its proximity to potential subscribers for that event. However, event delivery delay of that protocol is very long. Furthermore, continuous broadcasting messages in the network would cause large communication overhead and poor network performance.

In [12], Huang and Molina discuss a model with event sources, event brokers and event displayers, and modify it to be feasible in mobile networks. However, this method is based on the assumption that broker servers can always be organized into a multicast tree. An optimal Pub/Sub tree is proposed in [13] for routing events from the source to all interested recipients. Since each node knows its successors' subscriptions, flooding events can be avoided. However, the scalability of that approach is limited. As the network scale is growing or nodes' subscriptions change over time, tree maintenance incurs costs that are too high to be practical in the reality.

Moreover, EMMA [14] has focused on reliable event delivery by adopting an epidemic-style mechanism in a mobile ad hoc network. There are many unnecessary deliveries of events in that approach since that forwarding style does not consider the relationships of each node's subscriptions. Mottola *et al.* [15] propose a scheme to build and maintain event delivery structures for content-based routing in mobile ad hoc networks. They adopt and extend the MAODV (Multicast Ad hoc On-Demand Distance Vector) tree maintenance mechanism [16].

### 2.3. Routing Strategies in Pub/Sub Systems for DTN

Extending Pub/Sub systems in DTNs introduces new challenges because of the networks' intermittent connectivity. To the best of our knowledge, the only work on Pub/Sub communication in DTNs is discussed in [17,18]. The authors in [17] exploit distributed community detection from human traces and propose a Socio-Aware Overlay over detected communities for Pub/Sub communication. In that paper, centrality nodes are defined, with which an overlay is created. Since each node here needs to detect its own local community for the duration of the movement, the communication overhead is very high. This is particularly true for the centrality node. However, the sensors' energy is limited and, therefore, that strategy is not suitable for DTSNs.

In [18], the authors aim at Pub/Sub-based content distribution in DTNs, which achieves a more efficient utilization of network resources. Content is identified using a channel-based subscription system: interested users subscribe to channels and senders publish content by sending it as DTN bundles to the channel. Per-bundle utility calculation is deployed for local replication decisions and each node individually applies prioritization to control bundle processing. Finally, self-organized local prioritization can lead to a better overall performance in the network.

## 3. Network Model

**Figure 1.**
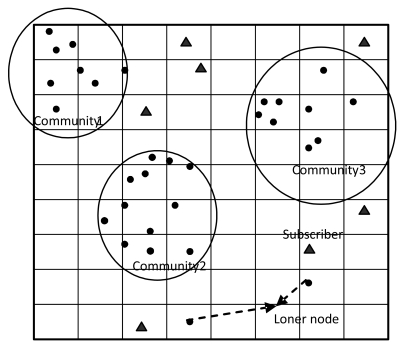
An example to illustrate communities of the network.

In this paper, nodes in the network fall into two categories: a large number of static sensor nodes and a small number of mobile nodes. Static sensor nodes work as publishers, while mobile nodes are special nodes (such as people, animals or vehicles) that work as subscribers. We assume that all sensor nodes are randomly deployed in a square region, which is divided into grids of identical size, as shown in Figure 1. Moreover, we further assume some additional characteristics in our modeling:

1. Each of the sensor nodes has a unique ID number.
2. The mobility of all the mobile subscriber nodes in the given area is assumed to follow the Random Waypoint Model (RWP) [19].
3. Since the energy of mobile subscribers can be complemented timely, we assume both the energy and the memory queue of the mobile subscribers are adequate.
4. Every node can directly communicate only with the nodes in the same gird or the nodes located in the neighboring grids sharing at least one side or one corner with the grid of this node.
5. The mobile subscriber nodes cannot directly communicate with each other.
6. All sensor nodes are aware of their locations.

In DTSN, each connective Graph formed by some sensor nodes in the network is called a community. Since the communication range of the sensor node is finite, the whole network is not connective and is divided into several communities as a result. We can see that only sensors in the same community can communicate with each other. Moreover, sensor nodes in the same community share the same community ID. An example of communities is shown in Figure 1, where all the sensors in the network are divided into three communities. However, there are also sensor nodes that do not belong to any communities in the network, named loner nodes.

## 4. The Proposed Community-Based Event Delivery Protocol

In this section, we present a novel event delivery protocol CED in Pub/Sub systems consisting of two components. CED aims to attain a high data delivery ratio with minimum data delivery overhead/delay by using both communities and mobility prediction of mobile subscriber nodes.

### 4.1. Routing Strategies in Pub/Sub Systems for DTN

In our network model, mobile subscribers are capable of continuously moving in different grids of the network. According to the RWP model, each subscriber is aware of its next destination location. Besides, mobile subscribers can easily obtain their locations from an attached extra device, for example a GPS. Thus, all the girds that a subscriber crosses can be easily acquired using the two aforementioned variables.

The implementation of the event delivery strategy is composed of two steps. First, we discuss the process of determining whether a sensor in a community can communicate directly with a mobile subscriber node or not based on the current moving path of the subscriber nodes acquired by using mobility prediction. Then we present the detail event delivery strategy. Without loss of generality, we consider the mobile subscriber node *i* and the community *N*. The process of finding sensor nodes which could communicate directly with subscriber *i* in community *N* is conducted into four phases:

- Initially, if the subscriber *i*, while moving, comes within one grid, it will broadcast Hello messages to all its neighboring sensor nodes. The neighboring sensor nodes send back their information, including community numbers, grid numbers and their ID numbers to subscriber *i* thereafter.
- Then, once subscriber node *i* finds out that it connects to community *N* for the first time, it sends out PATH messages including its current mobility path to its neighboring sensor nodes which belong to community *N*. After that, these PATH messages are broadcasted among nodes in community *N*.
- When subscriber *i* changes its moving path after connecting to community *N*, its current moving path should also be reported using the method mentioned in phase b.
- After receiving the PATH messages, each sensor node in community *N* would compare its location with each grid that the node *i* passes, to learn whether it could communicate directly with node *i* or not. Let set *Z* denote sensors that could communicate directly with subscriber *i* in community *N*.

**Figure 2.**
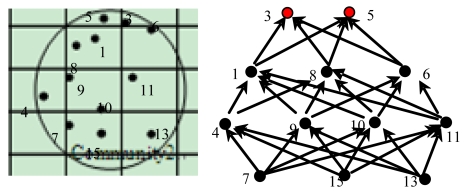
The routing graph of community 2.

After determining the set *Z* of community *N*, a routing graph is established based upon sensor nodes in set *Z*. Firstly, each senor node in set *Z* broadcasts its node ID message to all its adjacent sensor nodes except the nodes in set *Z*. For making an event delivery path towards the nodes in set *Z*, the sensor node which receives the node ID message will regard the message sender as its downstream relay node at first, and then it will repeat the process of nodes in set *Z* and broadcast its node ID message to all its neighboring sensor nodes. Other sensor nodes will repeat the above mentioned process until all the nodes in community N are included in the routing graph. Finally, a routing graph is established. An example of the constructed routing graph is illustrated in Figure 2, where we assume the set *Z* of community 2 in our network model contains sensor nodes with ID numbers of 3 and 5.

The event forwarding process is described as follows. Concretely, a sensor node forwards events of its own and those from the upstream nodes to its downstream. For sensor nodes that without upstream nodes, they just send their own events to the downstream. The sensors in the downstream will repeat this process until the events arrive at sensor nodes in set *Z*. Finally, the events will be delivered to subscriber *i* once the node in set *Z* communicates directly with subscriber *i*.

**Figure 3.**
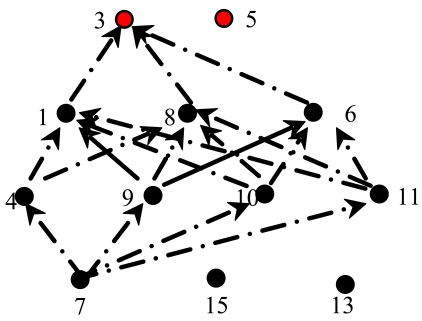
The transmission process from node 7 to node 3.

Since a sensor node may have multi-relay-sensors in its downstream, events in the source sensor node are evenly forwarded to its next relay sensors then, with the aim of balancing energy depletion among the relay sensor nodes. An example can be seen from Figure 3, where the event forwarding process from node 7 to node 3 in Figure 2 is presented.

Besides, when a subscriber node meets a loner node in its moving, events in the queue of the loner node are delivered directly to the subscriber node. Once a mobile subscriber node receives an event, it operates matching against its subscriptions immediately.

### 4.2. Queue Management

In opportunistic networks like DTSNs, in order to reach a certain data delivery ratio, queue management scheme is necessary. The goal of the queue management scheme is to properly arrange events, to determine event's delivery order in the buffer queue of sensor and to determine which event should be dropped when the queue is full. It is of great importance to the network performance. The main idea of our queue management scheme is to employ both the event survival time and the event successful delivery time to signify the amount of redundancy and the importance of a given event.

#### A. Event's survival time

Let's consider a sensor *j*; let C_j_ denote the clock of sensor j and let ξ_j_^m^ be the survival time of event m in the queue of sensor j. Here, we assume that the clocks in all sensors can be synchronized by current techniques to an acceptable accuracy. Thus, we can sketch our strategy for determining event's survival time as follows.

**Figure 4.**
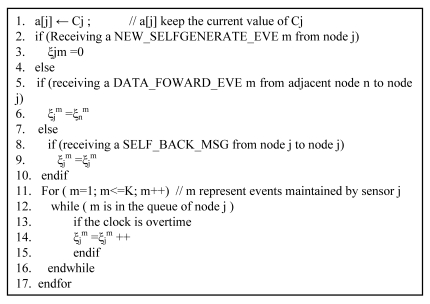
Pseudo-code of updating the survival time.

When an event is generated, its survival time is initialized to be zero. Whenever node *j* deliveries an event *m* to its 1-hop neighboring sensors, such as node *n*, the time used for transmitting could be ignored due to the short distance between the two nodes, thus the initial value of ξ_n_^m^ remains the same as ξ_i_^m^ before transmitting. As far as the source event which is inserted into source node's queue again after being transmitted to its next hop, its survival time is also assumed to be equal to the value before transmitting. Furthermore, for events maintained by sensors in the buffer queue, their survival time should be updated with the time clock. The general operations of this algorithm are presented in Figure 4, where parameter K denotes the maximum queue size of sensor.

#### B. Event's successful delivery time

We assume that a counter field is assigned in the event head to record the successful delivery time of an event. Upon generating an event, its successful delivery time is set to be zero. For the sake of simplicity, we prescribe once an event is delivered by its source sensor node, the successful delivery time of that event will be increased.

#### C. The implementation of queue management scheme

Each sensor will maintain a events record list coming from three sources.

- After the sensor acquires data from its sensing unit, it creates an event record and inserts the record into the data queue.
- When the sensor receives an event record from other sensors, it may insert the record into its data queue.
- After the sensor sends out an event record, it may insert the record again if this record is created by the source sensor node, because this record is not guaranteed to be delivered to all subscribers that have interest in it.

**Figure 5.**
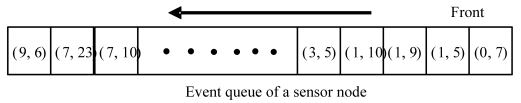
The arrangement of events in the data queue (where the number in the front represents event successful delivery time while the latter number represents event survival time).

Our queue management scheme is based on both the successful delivery time and the survival time of events (as shown in Figure 5) and we believe that the event with smaller successful delivery time is more important and should be transmitted with a higher priority. This is done by arranging the events in the queue with a decreasing order of their successful delivery time. Furthermore, for events with the same successful delivery time, priority should be given to those events that have smaller survival time. An event is dropped at the following two occasions. First, once the successful delivery time of an event is lager than a value, for example α, the event is dropped. Second, once the survival time of an event in the process of updating is larger than the network's delay tolerant threshold (the maximum delay value of events), the event is dropped. This is to reduce network energy consumption, given that the event either has been delivered to all its interested subscribers with a high probability or has been invalid in our application. Meanwhile, if the data queue of a sensor is full when an event arrives, no more events will be accepted.

## 5. Simulation Study

In this section, we simulate and evaluate two protocols: the proposed CED and the DG protocol under the mobility model stated in Section 3. In the DG protocol, events would be to only allow delivery when interested subscribers are in direct proximity to a sensor node.

### 5.1. Simulation Parameters

In our experimental environment, we define the event head of each event is composed of the following contents: {*A_1_= x_1_, A_2_= x_2_*}, where *A_1_, A_2_* are attribute names and *x_1_, x_2_* are double-type values randomly chosen from the range of (0, 10). Each subscriber has defined a subscription with the form of “*A_1_< x_1_Λ A_2_< x_2_*”, where *x1, x2* are also values randomly chosen from the range of (0, 10). In particular, we use the same data delivery ratio calculation method as in [20] to analyze the data delivery ratio in our simulation.

Moreover, we assume the data generation of each sensor follows a *poisson* process with an average arrival interval of 100 s. The network bandwidth is 10 kbps. The length of the whole test period is 2 hours, while over 200 seconds before the end of simulation, no event is generated. Other simulation parameters and their default values are summarized in Table 1. The performance metrics we use in our simulations are: data delivery ratio, data delivery delay, data delivery overhead and network lifetime. All the simulation results are averaged over 1,000 independent runs.

### 5.2. Performance Comparison

We compare the performance of the two protocols under the default parameters, with results presented in Table 2.

As we can see, the CED achieves a data delivery ratio of 86.5%, which is much higher than that of the DG protocol. This stems from the effective event delivery strategy where events information of sensors in a community are delivered when meeting with a mobile subscriber. Besides, the queue management scheme also ensures the credible transmission of events. In contrast, the DG protocol performs worse in terms of data delivery ratio, because the sensor nodes here can only transmit events to interested subscribers directly. Thus for sensors never meeting with some mobile subscribers, their events will never be received by interested subscribers.

In addition to the data delivery ratio, we are also interested in the data delivery delay and average copies. As shown in Table 2, the DG protocol has a longer data delivery delay than CED, for events in a sensor can only be forwarded to interested mobile subscribers directly. As a result, the transmission speed of events is low. On the other hand, due to the efficient event delivery strategy based on communities, the CED protocol outperforms DG in terms of data delivery delay. Moreover, In CED, as mentioned above, both the event successful delivery time and the event survival time are adopted to manage the data queue of sensors and thus invalid events are cleared away timely from the network. As a result, the number of average event copies in CED protocol is much lower than that in DG.

**Figure 6.**
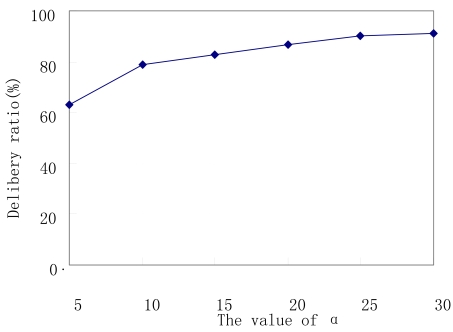
Delivery ratio vs value of α.

We also find out that the performance of CED varies with different values of event successful delivery time α. e.g. the data delivery ratios of CED varying with different values of α are shown in Figure 6. As we can see, the delivery ratio of CED increases with the increase of α. This is because with too small value of α, events in the sensors would be discarded only after very small times of successful delivery, resulting in the loss of some events before their being received by some relevant subscribers.

### 5.3. Impact of Varying Node Speed

**Figure 7.**
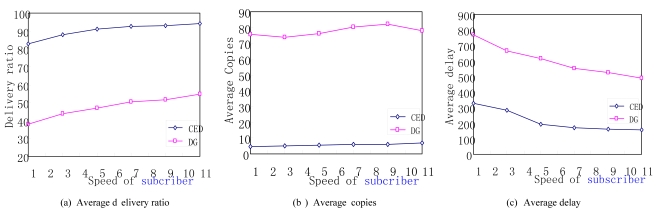
Impact of subscriber moving speed.

Network performance is closely related to node moving speed, so this group of experiments depicts the performance of the two protocols by varying the maximum moving speed of subscriber nodes. The experimental results can be seen from Figure 7.

We obtain that the CED protocol outperforms DG in terms of delivery ratio. With the increase of node movement speed, the delivery ratios of both CED and DG rise. This is because the subscriber node with a higher speed has a higher frequency of meeting sensors in a community. Thus, events have a higher chance to be delivered before they are dropped. We also notice that the transmission overhead of the proposed CED increases with the increase of node moving speed, as shown in Figure 7(b). This is because more event copies are produced when more sensors are encountered with higher node moving speed then. Figure 7(b) also depicts that the moving speed of subscribers has little impact on the average event copies in DG protocol. Figure 7(c) demonstrates that with the increase of node moving speed, the delivery delay of both CED and DG decreases. This is reasonable since events can be forwarded to interested subscribers quicker with a higher node moving speed.

### 5.4. Impact of Varying Sensor Node Density

**Figure 8.**
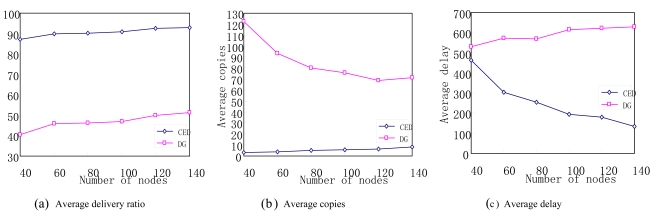
Impact of sensor node density.

Figure 8 illustrates the impact of sensor node density by varying the total number of sensor nodes in the network. As shown in Figure 8(a), the delivery ratios of both the two protocols vary slightly with the increase of sensor node density, which demonstrates that node density doesn't have a significant impact on data delivery ratios in both the CED and DG protocols. Meanwhile, we have also noticed that the CED protocol can always get higher data delivery ratio than the DG one with the increase of sensor node density. This is reasonable because the event delivery strategy is more aggressive in the CED protocol. Figure 8(b) depicts with higher sensor node density, the number of event copies in CED protocol rises while the duplicate event number decreases sharply in the DG protocol. This can be explained as following. In the CED protocol, more sensor nodes may enlarge the size of a community and make the total number of events in the community increase. Thus, more events can be delivered to a subscriber once the subscriber comes into contact with the community. Moreover, more events in the buffer queue of subscribers also make the number of re-forwarded events increase as a result. In the DG protocol, however, more sensors cannot meet the mobile subscribers in a timely way with more sensor nodes being deployed in the network. Thus events in these sensors are unlikely to be received by subscribers. This leads to the decrease of event copies in DG.

As more copies can enlarge the opportunity to deliver the matched events to their subscribers, the average data delivery delay decreases in the CED protocol with the increase of sensor node density. In contrast, the data delivery delay decreases in the DG protocol with higher sensor node density, as shown in Figure 8(c).

### 5.5. Analysis of Network Life

The *network lifetime* is defined as the duration from the very beginning of the network operation until the first sensor node dies. We observe that the DG protocol enjoys a longer network lifetime, since a sensor transmits events only when mobile subscribers come within the communication range of the sensor node, and thus much energy can be saved. However, the CED protocol maintains a shorter network lifetime. This is reasonable because more copies are generated and more events are transmitted in CED, thus producing more overhead. On the other hand, we can see from Table 3 that though the network lifetime of CED is shorter than DG, the data delivery ratio of CED is much higher than that in DG protocol, demonstrating that the proposed CED protocol can better deal with the tradeoff between the data delivery ratio and the delivery overhead than DG.

## 6. Conclusions

This paper deals with extending publish/subscriber systems in delay tolerant sensor networks. We propose CED, a community-based event delivery protocol in a Pub/Sub system tailored for DTSNs. The contributions of this paper are as follows:

1. We divide the network into several communities according to the connectivity of sensor nodes.
2. A dynamic routing mechanism was proposed, where events in a community are delivered to relevant mobile subscribers.
3. An effective queue management scheme was proposed. According to this scheme, events with too large successful delivery time or too long survival time should be dropped to make the full use of network bandwidth and to reduce network energy consumption.

## Figures and Tables

**Table 1.** Simulation parameters.

**Parameter**	**Default Value**
Network size	200 × 200
Number of Grids	15 × 15
Number of sensor node	100
Number of subscribe node	10
Initial energy of each sensor node (J)	10 J
Size of each event(bite)	250 bits
Number of events successfully transferred per second	20
E *_elec_*	50 nJ/bit
ε__fs_	10 pJ/bit/m^2^
ε__mp_	0.0013 pJ/bit/m^4^
Speed of subscribe node V(m/s)	0-5
Pause time Tpause (s)	0∼120
Maximum queue size of sensor	200 events
Value of α	20
Maximum delay tolerant value (s)	2,000 s
Threshold valueθ(J)	5
Value of γ	4

**Table 2.** Simulation results with default parameters.

	**CED**	**DG**
**Delivery ratio (%)**	86.5	47.0
**Average copies of each event**	4.2	75.9
**Average delay(s)**	230.6	615.5

**Table 3.** Network life using the four protocols.

	**CED**	**DG**
**Network lifetime(day)**	3.57	6.04
**Delivery ratio(%)**	86.5	47.0
